# Histology, 12p status, and IMP3 expression separate subtypes in testicular teratomas

**DOI:** 10.1007/s00428-020-02771-2

**Published:** 2020-03-06

**Authors:** Dávid Semjén, Krisztina Bíró, Emese Kapitány, Endre Kálmán, Tamás Tornóczky, Béla Kajtár

**Affiliations:** 1 Department of Pathology, Medical School and Clinical Centre, University of Pécs, Szigeti str. 12, Pécs, H7624 Hungary; 2 Department of Medical Oncology and Clinical Pharmacology “C”, National Institute of Oncology, Budapest, Hungary

**Keywords:** Testicular teratoma, FISH, 12p abnormality, IMP3, Prepubertal

## Abstract

**Introduction:**

Two types of testicular teratomas are distinguished by the current WHO classification. Prepubertal-type teratomas are benign, while postpubertal-type teratomas are considered malignant with metastatic potential, and are associated with germ cell neoplasia in situ. Prepubertal-type cases have been reported in the adult testis potentially causing confusion and overtreatment. Demonstration of the absence of 12p abnormalities with fluorescence in situ hybridization may facilitate diagnosis. Recently, IMP3 has emerged as a potential marker of malignancy in this context.

**Aims:**

The aim of this study was to assess histological characteristics, IMP3 expression and the presence of 12p abnormalities of pure testicular teratomas.

**Results:**

Thirty-seven cases were studied, 7 patients were children and 30 were adults. Six out of 7 pediatric cases showed no 12p abnormality and were IMP3 positive. Seventy-four percent and 79% of adult cases showed 12p abnormalities and IMP3 expression, respectively. Negative cases were not associated with in situ neoplasia or metastasis, they were smaller (mean, 14 vs 39 mm), showed less histological diversity (2.4 vs 4.0 types of tissues on average) compared to positive cases.

**Conclusion:**

Our study provides further evidence that prepubertal-type (type I) teratomas may appear in adult testes, thus teratomas in adults may be either benign (type I) or malignant (type II). IMP3 expression may aid the distinction between type I and type II teratomas of the postpubertal testis even when GCNIS and 12p status cannot be assessed.

**Electronic supplementary material:**

The online version of this article (10.1007/s00428-020-02771-2) contains supplementary material, which is available to authorized users.

## Introduction

Teratomas are germ cell tumors showing diverse histological differentiation comprising of at least two, usually all three different germinal layers (endoderm, ectoderm, and mesoderm). Teratomas may occur in a wide range of age groups; however, prepubertal- and postpubertal-type cases (type I and II, respectively) appear to show distinct genetic background and biological behavior [1]. Prepubertal-type teratomas typically behave as benign tumors and are not associated with chromosomal alterations involving the short arm of chromosome 12 (12p). These cases are generally pure teratomas and show no other germ cell tumor (GCT) components [2, 3]. Postpubertal-type teratomas are rarely pure tumors, they are regarded as malignant showing metastatic potential, the majority of them harbors 12p abnormalities typical of malignant germ cell tumors [4, 5, 6]. Germ cell neoplasia in situ (GCNIS) is virtually always present in the adjacent testicular tissue, a finding that is lacking in prepubertal-type cases [7]. Despite the name suggesting otherwise, prepubertal-type teratomas are not limited to testes of children, these benign teratomas may occur in adults beyond puberty [8, 9, 10]. In fact, the absolute number of adult cases exceeds that of pediatric cases according to our own data [11].

12p abnormalities result in excess DNA of the short arm of chromosome 12 either in the form of *isochromosome 12p*—i(12p)—or other forms of copy number gains of 12p segments dubbed *12p overrepresentation* or *12p gain* [5]. These abnormalities were first demonstrated using karyotyping [5], however, they may be demonstrated with fluorescence in situ hybridization (FISH) even using formalin-fixed, paraffin-embedded tissues.

Recently, it was suggested that IMP3 labeling of tumor cells may be an indication of malignant behavior. IMP3 is the third member of the IGF-2 mRNA-binding protein family [12]. IMP3 is expressed in embryonic tissues physiologically and plays an important role in cell migration and early embryogenesis, and may be detected in oocytes and granulosa cells in the ovaries as well as in spermatogonia, spermatocytes, and spermatozoa in the testes [13]. According to some studies IMP3 is not expressed in benign tissues, acts as an oncoprotein triggering growth, invasion and metastasis in malignant tumors [14]. Based on literature data, IMP3 seems to play a role in the development of GCTs and can be detected with immunohistochemistry in almost all testicular GCTs [13] with positive staining in both primary and metastatic teratomas in adults [15].

The aim of the present study was to determine the characteristics of prepubertal-type and postpubertal-type pure testicular teratomas regarding histological composition, IMP3 expression, and the presence of 12p abnormalities.

## Materials and methods

### Patients and samples

Six hundred fifty-five testicular germ cell tumors submitted for histological analysis between 1998 and 2019 at the Department of Pathology (University of Pécs, Medical School) have been reviewed for this study. Fifteen cases showing pure testicular teratoma were available for further investigations. Twenty-two additional cases between 2000 and 2016 with available archived samples were selected from the archives of the National Institute of Oncology, Hungary. Clinicopathological information of the 37 total cases are depicted in Table 1. Approval for research was requested and received from the Ethics Committee (21679-2/2016/EKU as well as PTE 47407/2017).

**Table 1:** Clinicopathological information of testicular teratoma cases

Case ID	Age (years)	Size (mm)	RPLND	Chemotherapy	LN met	Follow-up (months)
#1	< 1 (11 months)	33	–	–	–	89
#2	< 1 (4 months)	30	–	–	–	43
#3	< 1 (6 months)	20	–	–	–	114
#4	< 1 (8 months)	20	–	–	–	89
#5	< 1 (4 months)	15	–	–	–	52
#6	< 1 (9 months)	15	–	–	–	1
#7	5	26	–	–	–	23
#8	18	13	+	–	–	97
#9	20	22	–	–	–	105
#10	27	8	–	–	–	55
#11	30	15	–	–	–	63
#12	33	20	–	–	–	88
#13	36	10	–	–	–	36
#14	38	10	ND	ND	ND	98
#15	25	25	+	+	+	221
#16	21	25	–	–	–	81
#17	32	22	+	–	+	167
#18	41	9	+	+	+	173
#19	21	30	–	–	–	166
#20	22	20	+	+	–	207
#21	38	60	+	+	+	53
#22	22	30	+	–	–	177
#23	29	19	+	–	+	118
#24	29	22	–	–	–	64
#25	18	85	+	–	–	61
#26	18	38	–	–	–	67
#27	24	14	–	–	–	53
#28	22	35	–	–	–	65
#29	38	60	+	+	+	53
#30	31	40	+	+	+	211*
#31	43	32	+	+	+	65
#32	26	18	+	+	+	36*
#33	36	36	+	+	+	52
#34	25	35	+	+	+	66
#35	34	110	+	+	+	90
#36	33	70	+	+	+	123
#37	19	54	–	+	–	182

#1–7, pediatric cases; #8–37 cases, adult cases

*ND*, no data; *RPLND*, retroperitoneal lymph node dissection; *LN met*, lymph node metastasis

*Patient died

### Immunohistochemistry

Freshly cut, 4-μm thick sections of formalin-fixed, paraffin-embedded (FFPE) tissue blocks were analyzed, the entire tumor tissue was sampled in each case. IMP3 expression was detected with a mouse monoclonal anti-human antibody (clone 69.1; Dako, Glostrup, Denmark) in a dilution of 1:100 after peroxidase blocking with H_2_O_2_ (Dako) for 10 min. When the presence or absence of GCNIS was not evident, placental alkaline phosphatase (PLAP) expression was examined using a mouse monoclonal anti-human antibody (clone 8A9, Dako) in a dilution of 1:40. High-temperature pretreatment of slides was carried out in an autoclave with citrate buffer, pH 9.0 for 20 min. The Envision system (Dako) was used to visualize the staining. When present in the samples, seminiferous tubules containing spermatogonia were used as positive controls for IMP3 expression. A case was considered positive if staining of any intensity was observed, negative when no staining was present. Ten tissue samples showing non-neoplastic epithelium from urinary tract, gastrointestinal tract as well as skin were tested for IMP3 expression as controls (Electronic Supplemental Material 1).

### Fluorescence in situ hybridization

Four-micrometer thick sections were cut from FFPE tissue blocks. Following deparaffination using xylol, enzymatic and heat pretreatment was performed using SPOT-Light™ Tissue Pretreatment Kit (Zymed-Invitrogen-Thermo Fisher Scientific) following instructions of the manufacturer. A probe mix containing SpectrumGreen labeled *ETV6* (12p13), SpectrumOrange labeled *RUNX1* (21q22) (LSI ETV6-RUNX1 probe kit, Abbott-Vysis, USA) and SpectrumAqua labeled CEP12 was applied and hybridization was performed overnight. Stringency washing was performed following instructions of the manufacturer of the LSI ETV6-RUNX1 probe kit. The CEP12 probe was available from our own laboratory as described previously [16]. Signal counts of 12p and CEP12 FISH probes were obtained in two separate fluorescence channels individually of at least 50 cells per sample. To avoid contamination by non-neoplastic stromal and inflammatory cells, epithelial cells of cysts were investigated. Whenever it was possible, cells of different cysts were included. In some cases, recognizable chondroid tissue was assessed as well. Spot counting of individual cells was not always possible due to truncation and overlapping of nuclei. The following data were acquired for each sample: (1) mean count of 12p and CEP12, (2) percentage of cells with higher 12p than CEP12 signal counts, (3) percentage of cells with CEP12 signal count higher than 2, and (4) mean of 12p/CEP12 signal count ratio. Six cases of mixed non-seminomatous adult testicular tumors were used as positive, and four non-neoplastic tissue samples composed of squamous and glandular epithelium were used as negative controls. All of the positive controls showed polysomy of chromosome 12 with CEP12 spot counts above 2 in 68% of cells on average. Very few cells showed FISH signal patterns typical of i(12p), however, the number of 12p FISH spots exceeded CEP12 signal counts with a mean 12p/CEP12 ratio of 1.8 (1.3–2.5), and with 78% of cells showing higher 12p spot count than CEP12 on average. Negative controls showed a mean 12p/CEP12 ratio of 1.0, while 15% of cells showed more 12p signals than CEP12 on average. However, no 12p abnormality was present within these normal samples, and cells with lower 12p than CEP12 signal counts appeared with a similar frequency (Electronic Supplemental Material, Table 2). There was a clear separation of positive and negative controls using cut-off values defined as mean ± 2 × standard deviation (positive controls ≥ 41%, negative controls < 30%). The cut-off value for polysomy 12 was 12%.

**Table 2:** Histological characteristics of testicular teratomas

	Number of cases	Mean tumor size (mm)	GCNIS (%)	IMP3+ (%)	Squamous epithelium	Intestinal epithelium	Other epithelium	Neural tissue	Chondroid tissue	Bone tissue	Mesenchymal tissue	Mean number of different components
All cases	37	31	46	81	23	22	29	9	16	8	37	3.8
Prepubertal	7	23	0	86	4	6	5	3	4	4^d^	7	4.7
Postpubertal												
Total	30	33	57	79	19	16	24	6	12	4	30	3.6
Benign	7	14^a^	0	0	3	2	3^b^	0	0^c^	2	7	2.4
Malignant	23	39^a^	76	100	16	14	22^b^	6	12^c^	2^d^	23	4.0
Prepubertal cases by Cornejo et al.[ref]	11	20	0	46	8	10	5	5	5^e^	5^e^	5	NA
Postpubertal benign cases by Zhang et al. [ref]	25	23	0	NA	15	6	16	2	2	1	1	2.7

The table shows the cases with the presence of different histological components as well as the mean tumor size. Other epithelium indicates epithelial tissue different from those that are listed separately, and includes respiratory epithelium, seromucinous glands, as well as immature epithelium. Mesenchymal tissue represents muscle or adipose tissue as well as immature mesenchymal tissues. Benign postpubertal teratomas were defined as lack of GCNIS and 12p abnormality by FISH (type I teratoma), malignant cases showed GCNIS and or 12p abnormality (type II). Significant difference was detected between pairs marked with superscript: ^a^benign vs malignant postpubertal cases, mean tumor size: *p* < 0.001, ^b^appearance of other epithelium, benign vs malignant postpubertal cases: *p* = 0.006, ^c^appearance of cartilage, benign vs malignant postpubertal cases: *p* = 0.016, ^d^appearance of bone tissue, prepubertal vs malignant postpubertal cases: *p* = 0.0157. ^e^Cornejo et al. described 5 cases with cartilage and/or bone together. *NA*, data not available

## Results

Out of 37 pure testicular teratomas studied, seven were pediatric with a mean age of 14.6 months (4 months–5 years), and 30 were adult cases (mean age 28, 18–43 years). All pediatric cases were considered benign, retroperitoneal lymph node dissection (RPLND) was not performed, chemotherapy was not administered in these cases, and none showed lymph node metastasis. Clinical data of 29 adult cases were available; seventeen patients underwent RPLND, thirteen received chemotherapy, and thirteen had evidence of lymph node metastasis (Table 1).

### IMP3 expression and GCNIS

**Fig. 1:**
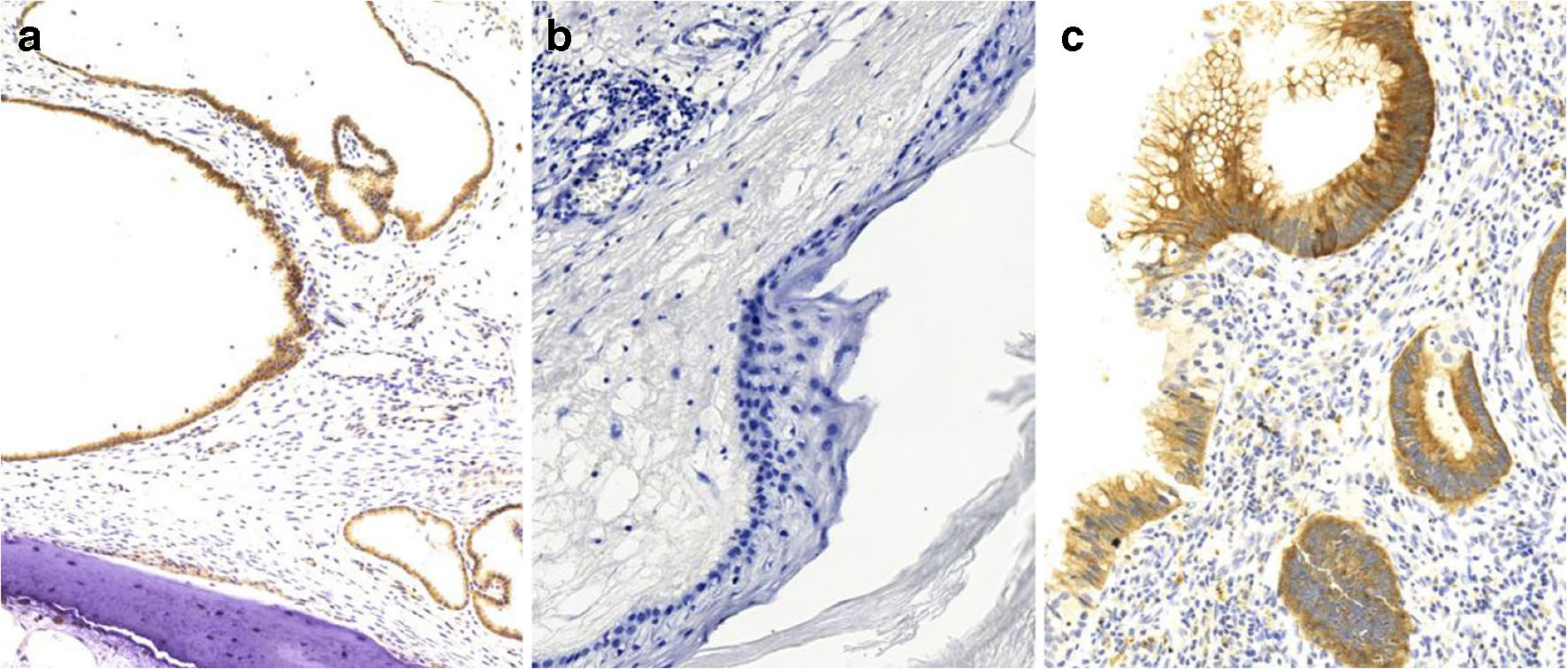
IMP3 expression in teratomas. Digital images show a magnification equivalent of × 100. Brown staining indicates IMP3 expression. **a** A pediatric case (ID#4) with strong to moderate expression of IMP3 in epithelial tissues. **b** A postpubertal case (ID#7) without IMP3 expression. **c** An adult case (ID#32) with strong expression of IMP3 in epithelial tissues

All thirteen adult cases with lymph node metastasis based on histological and/or radiological findings showed at least focal and weak IMP3 positivity in epithelial components, while 10/16 cases without metastasis showed IMP3 expression (one case was not available for analysis). IMP3 expression intensity and extent were heterogeneous ranging from weak to strong as well as from partial to complete, with 15–100% of cells showing staining. Few cases with metastasis demonstrated IMP3 labeling of mesenchymal tissues as well, however, IMP3 expression was mostly observed in epithelial tissues (Fig. 1). One of 13 adult cases with metastasis showed no seminiferous tubules making an evaluation of GCNIS impossible. Eight out of the 12 remaining cases showed the presence of GCNIS, four presented tubules containing Sertoli cells only. Fifteen of 16 adult cases without metastasis were available for GCNIS analysis, and 8 showed positivity. None of the pediatric cases showed GCNIS and six showed moderate to strong expression of IMP3 confined mostly to epithelial tissues. A case of a 5-year-old boy did not show any IMP3 positivity (Tables 1 and 2 and Electronic Supplemental Material 3).

### 12p abnormality as detected by FISH

**Fig. 2:**
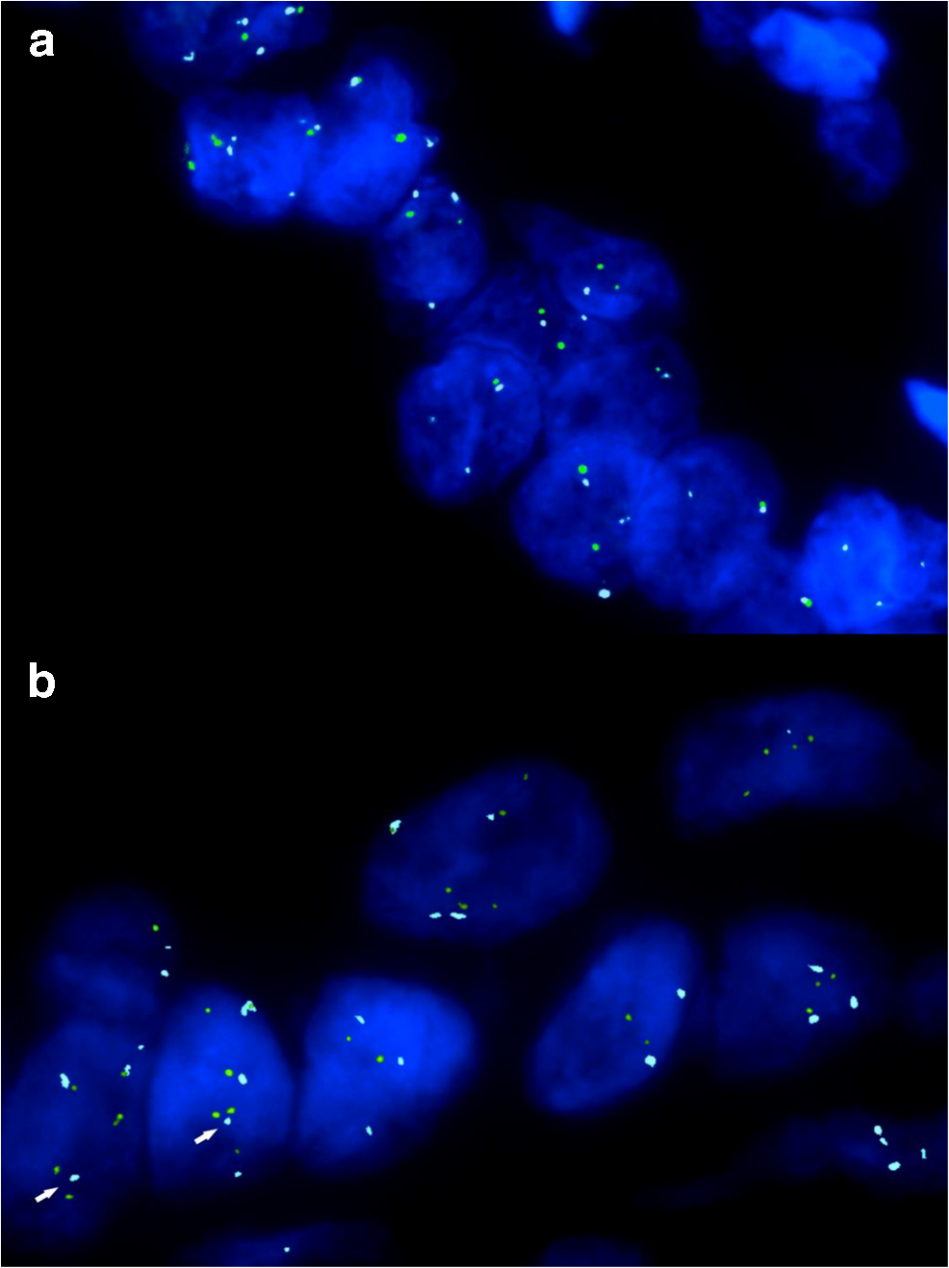
FISH images with and without 12p abnormality. Green, ETV6 FISH probe; Cyan, CEP12 FISH probe. Orange signals of RUNX1 probe were omitted. **a** Same count of green and cyan spots are present in the majority of cells. Signal counts appear to be higher than 2 in few nuclei due to overlapping. There is no indication of 12p gain. **b** Cells show polysomy with more than 2 signals for each FISH probe. The number of green signals exceed that of the cyan signals with two cells showing the typical FISH pattern indicating i(12p) (white arrows)

None of the 7 pediatric cases showed 12p gain or polysomy 12 based on FISH data. The mean percentage of cells with more than two CEP12 FISH signals, the 12p/CEP12 ratio as well as the percentage of cells with 12p > CEP12 FISH signal counts was similar to that of negative controls, the cut-off levels were not exceeded. Adult cases with metastasis showed 12p gain in eleven out of 11; in two cases, FISH was not informative due to weak FISH signals and high background fluorescence. With the exception of one, all cases with 12p gain showed polysomy 12 as well. Out of the 17 remaining cases, FISH was unsuccessful in one, 12p gain was positive in nine, while polysomy 12 was seen in eight cases (Fig. 2). All cases without 12p gain showed no IMP3 staining. The mean 12p/CEP12 ratio of the 12p gain positive cases was 1.9, four cases showed values below 1.5 (Electronic Supplemental Material 2).

### Histopathological characteristics

Histological characteristics of adult cases with metastasis were very similar to cases without metastasis but with IMP3 expression and 12p abnormality and differed from cases without such parameters. Thus, adult cases with IMP3 and 12p positivity were grouped together as postpubertal, malignant (type II), the others as postpubertal, benign (type I) (Table 2). The mean tumor size was different in the distinct teratoma types. On average, 23 (15–33) mm was measured in pediatric cases and 33 (8–110) mm in adult cases. Cases with metastasis showed larger size compared to cases without it, with a mean of 41 (9–110) mm for the previous group. When cases without metastasis were separated based on IMP3 expression and 12p abnormality, positive cases appeared similar to cases with metastasis, while negative cases were significantly smaller. The mean size was 14 (8–22) mm for IMP3/12p negative and 39 (9–110) mm for IMP3/12p positive cases (Student’s *t* test, *p* < 0.001).

The appearance of different tissue types was also analyzed. All cases showed mesenchymal tissues along with at least one type of epithelium with the exception of a single case that showed only mesenchymal tissues (Electronic Supplemental Material 3). The frequency of different tissue types showed important differences. Pediatric cases were most diverse with 4.7 different tissue components on average, while 4.0 and 2.4 components were seen in adult cases with or without IMP3 expression and 12p abnormality, respectively (Table 2 and Electronic Supplemental Material 3). The difference between the two latter groups reached statistical significance (Student’s *t* test, *p* < 0.001).

Pediatric cases were similar to adult cases with IMP3/12p positivity regarding tissue composition with one notable, significant difference: bone was frequent among the former and was rare in the latter group (Fisher’s exact test, *p* = 0.0157). Pediatric cases also showed difference compared with adults, IMP3/12p negative cases; epithelium other than squamous, neural tissue, chondroid, and osseous tissues were common among the former and rare among the latter, however, statistical significance was not reached. Adult cases with and without IMP3 expression and/or 12p abnormality showed significant differences in the occurrence of chondroid tissue as well as epithelial tissue other than squamous or intestinal (Fisher’s exact test, *p* = 0.016 and 0.006, respectively). Seventeen patients underwent RPLND, 13 received chemotherapy. Follow-up data are limited, after a median of 103 months, only two patients died.

## Discussion

Distinction between teratomas with and without malignant potential is vital, since the former often requires retroperitoneal lymph node dissection as well as chemotherapy, while the latter should be regarded benign and testis-sparing surgery may be considered with small tumors [17, 18]. The prepubertal- and postpubertal-type teratomas, as defined by the current WHO classification of testicular, tumors mostly correspond to benign and malignant tumors, respectively. The former is characterized by the absence of GCNIS and 12p abnormalities, while the latter typically shows these features. However, prepubertal-type teratoma may appear in postpubertal testes [7–10].

12p abnormalities are associated with gain of genetic material corresponding to the short arm of chromosome 12 usually with polysomy. These structural cytogenetic abnormalities may be detected using dual-color FISH probes even on FFPE slides based on the number and position of different FISH signals. Isochromosome of 12p, the commonest of 12p abnormalities may be detected when there is an extra 12p FISH signal in the proximity to one of the centromere probes [19]. However, orientation and truncation of the nuclei in a section significantly influence the recognition of this pattern. Many investigators have found that this signal pattern was seen only in 2–5% of tumor cells, when i(12p) was positive [6]. Several studies have focused on detecting overrepresentation of 12p instead, which was often defined as the ratio of 12p/CEP12 FISH signals per nuclei being over 1.5 [6, 20]. This cut-off value, however, does not take the effects of nuclear truncation into consideration that may distort the average FISH signal counts within the sample. As a result, the mean ratio may be below 1.5 even in i(12p) positive cell lines [21].

In our study, a commercially available FISH probe for 12p13 was used, the same that was used by *Velagaleti* et al. to detect i(12p) [22]. The typical signal pattern of i(12p) was detected only in a minority of cases, however, cases with 12p abnormality were distinguished from negative ones by a cut-off value of more than 30% of cells showing more 12p than CEP12 FISH signals in cells. Evaluable FISH signals were seen in 92% of cases, all metastatic teratomas showed the presence of 12p abnormality, while pediatric cases were negative. 12p copy number aberrations may be investigated by other molecular techniques, such as multiple ligation probe amplification (MLPA) and next-generation sequencing (NGS). However, percentage of tumor cells is often low in the samples investigated increasing the likelihood of false negativity when in situ detection is not available.

IMP3 was described as a potential marker of malignant tumors since it is not expressed by benign tumors and is seen only in few normal adult tissues and in specific cell types [23]. We have detected IMP3 expression in all evaluable cases with metastasis and none of the adult cases without 12p abnormalities. However, IMP3 expression was found in six of 7 pediatric cases. Cornejo et al. have described IMP3 staining in 46% of pure testicular teratoma cases [5]. Interestingly, IMP3 positive cases in their and our study were all under the age of 14 months, while all cases above that age showed no staining. Information is not available in the literature regarding expression of IMP3 in normal tissues of human infants. This led us to investigate IMP3 expression with immunohistochemistry using various non-neoplastic epithelial tissues from 10 male patients below the age of four. IMP3 expression was consistently absent over and present below the age of 14 months (Electronic Supplemental Material 3). These findings need further validation in a larger cohort of samples, specifically, because only a single prepubertal case above the age of 4 was investigated in the present study, however, the findings suggest that IMP3 expression may be used as an indication of malignant potential only after the age of 14 months. Interpretation of IMP3 expression requires caution since expression is usually limited to epithelial tissues and may be only focal. With appropriate methodology aided by the use of positive and negative controls, preferably both internal and external, a positive reaction with IMP3 antibodies in any part of a teratoma raises the suspicion of postpubertal-type (malignant) in adults.

In our study of 37 testicular teratomas, pediatric cases showed no 12p abnormalities, no GCNIS, and had the most diverse appearance with all tissue types being frequent. The size was intermediate compared to the other two types (23 mm on average). IMP3 expression was seen before the age of 14 months in all cases investigated. Malignant adult teratomas showed IMP3 expression as well as 12p abnormalities. GCNIS was present in 76% of evaluable cases. Histological composition showed similar diversity to pediatric cases, however, bone appeared significantly less frequently. Tumor size was the largest (39 mm on average), 57% of cases were associated with histological or radiological diagnosis of lymph node metastasis. Approximately, a quarter of adult teratomas (7/30) showed no IMP3 expression or 12p abnormality and were not associated with GCNIS. These tumors were the smallest (14 mm on average), showed the least diversity with only 2.4 different tissue components on average. Neural tissue and cartilage were significantly less frequent compared with the other teratoma types. None of these adult cases showed evidence of metastasis, after a median 7-year follow up, none of the patients died.

Zhang et al. described 25 patients older than 12 with pure testicular teratomas without 12p abnormalities [8]. Squamous epithelium was found in twenty cases, half of which appeared as dermoid cyst. On average, 2.7 different tissue components were described, similar to 2.4 in our series. The frequency of different components was comparable to our findings, however, mean size was 23 mm, larger than in our series, comparable to what we have found in our pediatric cases (Table 2). Neural tissue, as well as cartilage, was rare, similar to our observations.

The above findings are consistent with type I and type II teratomas of the testis. The type I teratomas are benign tumors, which typically occur in the prepubertal testis, and may, however, also occur in the postpubertal testis. The type II teratomas are malignant and occur only in the postpubertal testis (Table 3) [1, 7, 19]. Postpubertal benign teratomas may in fact be prepubertal lesions recognized late. The smaller size and reduced histological diversity may reflect failed involution; however, there are no reports of spontaneous disappearance of pediatric teratomas. Additionally, the mean age at diagnosis of postpubertal benign teratomas was 29, far exceeding the mean age of 14.6 months of patients with prepubertal lesions in our study.

**Table 3:** Characteristics of proposed testicular teratoma types

Teratoma type	Age (years)^a^	GCNIS	IMP3	12p+	Size (mm)^a^	Histologic features
Prepubertal	0–3	No	Yes	No	15–31	All components are frequent
Postpubertal benign^b^	22–36	No	No	No	9–19	Cartilage, bone, neural and other epithelial tissue are rare
Postpubertal malignant^c^	25–31	Yes	Yes	Yes	28–49	Bone tissue is rare, other components frequent

GCNIS, germ cell neoplasia in situ. 12p+: 12p abnormality detected by FISH. ^a^95% confidence interval of age and tumor size are shown. ^b^WHO prepubertal type teratoma in postpubertal testis, ^c^WHO postpubertal type teratoma in postpubertal testis

Based on the classification of germ cell tumors proposed by Oosterhuis et al., two distinct types of testicular teratomas may be defined [7]. Prepubertal-type teratomas are defined as type I with no 12p aberrations, diploid karyotype, and benign behavior. Postpubertal malignant teratomas are described as type II with frequent gains of 12p as well as aneuploid karyotype. Ovarian dermoid cysts are defined as type IV. Type I tumors reported by Oosterhuis et al. occur rarely in the postpubertal testis and are suggested to either represent late recognition of a prepubertal lesion, or to be derived of a dormant germ cell with developmental potential intermediate between type I and type IV tumors [7].

In summary, our study provides further evidence that prepubertal-type teratomas may appear in adult testes, thus teratomas may be benign or malignant in that age group and terminology should emphasize the distinction to aid clinical management. Organ-sparing surgery may be considered in cases of benign adult teratoma of prepubertal-type, and lymph node dissection and chemotherapy may be avoided. Our findings raise the possibility that IMP3 expression may be effectively used to distinguish teratomas of benign versus malignant potential, even when FISH studies are not available or are unsuccessful; however, a validation study on a large, multi-institutional cohort is necessary for verification. In our series, 10% of adult cases were not evaluable by FISH (3/30), and it was not possible to assess GCNIS in 20% (6/30). However, the distinction cannot be made before the age of 14 months when IMP3 expression may be present even in normal tissues.

## Electronic supplementary material

ESM 1 (DOCX 12 kb)

ESM 2 (DOCX 18 kb)

ESM 3 (DOCX 17 kb)
