# Supplementary material for: Histology, 12p status, and IMP3 expression separate subtypes in testicular teratomas
Source: Virchows Arch. 2020 Mar 6;477(1):103–10. doi: 10.1007/s00428-020-02771-2 (PMC7320034; doi:10.1007/s00428-020-02771-2)
Supplement: Supplementary file 1 — (DOCX 12 kb) [file 428_2020_2771_MOESM1_ESM.docx]

Electronic Supplemental Material, Table 1 IMP3 expression in normal, pediatric tissues

| ID | Age (months) | Tissue | IMP3 |
| --- | --- | --- | --- |
| I1 | < 1 | colon | positive |
| I2 | < 1 | ureter | positive |
| I3 | 4 | ileum | positive |
| I4 | 4 | epidermis | positive |
| I5 | 15 | ureter | negative |
| I6 | 15 | dermoid cyst (skin) | negative |
| I7 | 23 | renal pelvis | negative |
| I8 | 31 | colon | negative |
| I9 | 36 | appendix | negative |
| I10 | 48 | duodenum | negative |
