# Supplementary material for: Histology, 12p status, and IMP3 expression separate subtypes in testicular teratomas
Source: Virchows Arch. 2020 Mar 6;477(1):103–10. doi: 10.1007/s00428-020-02771-2 (PMC7320034; doi:10.1007/s00428-020-02771-2)
Supplement: Supplementary file 2 — (DOCX 18 kb) [file 428_2020_2771_MOESM2_ESM.docx]

Electronic Supplemental Material, Table 2 Detailed FISH results of testicular teratoma cases

| Case ID | 12p | CEP12 | 12p/CEP12 ratio | 12p>CEP12 (%) | 12p<CEP12 (%) | polysomy 12 (%) |  | Case ID | 12p | CEP12 | 12p/CEP12 ratio | 12p>CEP12 (%) | 12p<CEP12 (%) | polysomy 12 (%) |
| --- | --- | --- | --- | --- | --- | --- | --- | --- | --- | --- | --- | --- | --- | --- |
| P1 | 6.0 | 4.4 | 1.4 | 76 | 0 | 84 |  | 15 | 3.5 | 2.7 | 1.3 | 40 | 2 | 48 |
| P2 | 4.1 | 2.6 | 1.7 | 84 | 8 | 56 |  | 16 | 3.2 | 2.1 | 1.8 | 82 | 0 | 40 |
| P3 | 5.0 | 2.2 | 2.5 | 96 | 0 | 24 |  | 17 | 4.6 | 2.6 | 2.0 | 92 | 0 | 60 |
| P4 | 6.9 | 4.1 | 1.9 | 78 | 0 | 86 |  | 18 | 3.5 | 2.8 | 1.3 | 46 | 0 | 50 |
| P5 | 6.0 | 3.0 | 2.1 | 92 | 4 | 68 |  | 19 | 5.0 | 3.5 | 1.5 | 84 | 12 | 78 |
| P6 | 4.4 | 4.0 | 1.3 | 44 | 12 | 88 |  | 20 | 4.6 | 3.6 | 1.3 | 50 | 0 | 64 |
| N1 | 1.7 | 1.6 | 1.1 | 14 | 8 | 0 |  | 21 | 3.1 | 2.0 | 1.6 | 54 | 6 | 12 |
| N2 | 1.7 | 1.8 | 1.0 | 20 | 20 | 8 |  | 22 | 4.0 | 2.7 | 1.7 | 64 | 4 | 60 |
| N3 | 1.6 | 1.8 | 0.9 | 4 | 4 | 4 |  | 23 | 4.1 | 2.3 | 2.0 | 72 | 0 | 36 |
| N4 | 1.8 | 1.8 | 1.1 | 20 | 12 | 0 |  | 24 | 5.3 | 3.2 | 1.8 | 84 | 0 | 68 |
| 1 | 2.1 | 2.1 | 1.0 | 0 | 0 | 8 |  | 25 | 3.8 | 2.8 | 1.4 | 52 | 0 | 52 |
| 2 | 1.9 | 1.8 | 1.1 | 10 | 0 | 8 |  | 26 | 5.5 | 3.0 | 1.9 | 96 | 0 | 64 |
| 3 | 2.0 | 2.0 | 1.0 | 0 | 0 | 0 |  | 27 | 4.6 | 1.8 | 2.7 | 100 | 0 | 8 |
| 4 | 2.0 | 2.0 | 1.0 | 0 | 0 | 0 |  | 28 | 5.4 | 2.6 | 2.3 | 100 | 0 | 40 |
| 5 | 2.0 | 2.0 | 1.0 | 0 | 0 | 0 |  | 29 | 7.8 | 2.8 | 2.9 | 100 | 0 | 60 |
| 6 | 2.0 | 2.0 | 1.0 | 0 | 0 | 0 |  | 30 | 5.8 | 2.9 | 2.3 | 100 | 0 | 52 |
| 7 | 2.0 | 2.0 | 1.0 | 0 | 0 | 0 |  | 31 | 5.4 | 3.7 | 1.6 | 100 | 0 | 80 |
| 8 | 1.8 | 1.8 | 1.2 | 24 | 16 | 0 |  | 32 | 3.4 | 2.6 | 1.4 | 68 | 8 | 52 |
| 9 | 2.2 | 2.2 | 1.0 | 0 | 0 | 12 |  | 33 | 5.8 | 2.3 | 2.9 | 96 | 0 | 36 |
| 10 | 2.0 | 2.0 | 1.0 | 0 | 0 | 0 |  | 34 | 6.0 | 2.1 | 3.1 | 100 | 0 | 20 |
| 11 | 2.0 | 2.0 | 1.1 | 10 | 8 | 12 |  | 35 | ND |  |  |  |  |  |
| 12 | 2.1 | 2.1 | 1.0 | 0 | 0 | 6 |  | 36 | ND |  |  |  |  |  |
| 13 | 2.0 | 2.0 | 1.0 | 0 | 0 | 0 |  | 37 | ND |  |  |  |  |  |
| 14 | 1.2 | 1.2 | 1.0 | 8 | 8 | 0 |  |  |  |  |  |  |  |  |

Case ID: P1-6: positive controls, N1-4: negative controls, 1-7: prepubertal cases, 8-37: postpubertal cases, 12p: Mean number of FISH spots per nucleus corresponding to 12p (ETV6), CEP12: Mean number of FISH spots per cell corresponding to centromere of chromosome 12, 12p/CEP12 ratio: Mean ratio of 12p and CEP12 FISH spot numbers per cell, 12p>CEP12 (%): percentage of cells with more 12p than CEP12 FISH spots, 12p<CEP12 (%): percentage of cells with less 12p than CEP12 FISH spots, Polysomy 12 (%): Percentage of cells with CEP12 FISH spot number higher than 2.
