# Supplementary material for: Histology, 12p status, and IMP3 expression separate subtypes in testicular teratomas
Source: Virchows Arch. 2020 Mar 6;477(1):103–10. doi: 10.1007/s00428-020-02771-2 (PMC7320034; doi:10.1007/s00428-020-02771-2)
Supplement: Supplementary file 3 — (DOCX 17 kb) [file 428_2020_2771_MOESM3_ESM.docx]

Electronic Supplemental Material, Table 3 Detailed histological characteristics of testicular teratoma cases.

| Cae ID | Age (years) | Mean tumor size (mm) | 12p gain | GCNIS | IMP3 expression | Squamous epithelium | Intestinal epithelium | Other eithelium | Neural tissue | Chondroid tissue | Bone tissue | Mesenchymal tissue |
| --- | --- | --- | --- | --- | --- | --- | --- | --- | --- | --- | --- | --- |
| 1 | <1 | 33 | - | - | + | + | + | + | + | + | + | + |
| 2 | <1 | 30 | - | - | + | - | + | + | - | + | - | + |
| 3 | <1 | 20 | - | - | + | + | - | + | + | + | + | + |
| 4 | <1 | 20 | - | - | + | - | + | + | + | + | + | + |
| 5 | <1 | 15 | - | - | + | - | + | - | - | - | - | + |
| 6 | < 1 | 15 | - | - | + | + | + | - | - | - | - | + |
| 7 | 5 | 26 | - | - | - | + | + | + | - | - | + | + |
| 8 | 18 | 13 | - | -^a^ | - | + | - | + | - | - | - | + |
| 9 | 20 | 22 | - | -^a^ | - | - | + | - | - | - | - | + |
| 10 | 27 | 8 | - | -^a^ | - | + | + | - | - | - | - | + |
| 11 | 30 | 15 | - | -^a^ | - | - | - | + | - | - | - | + |
| 12 | 33 | 20 | - | -^a^ | - | - | - | + | - | - | - | + |
| 13 | 36 | 10 | - | -^a^ | - | + | - | - | - | - | + | + |
| 14 | 38 | 10 | - | -^a^ | NI | - | - | - | - | - | + | + |
| 15 | 25 | 25 | + | NI | + | + | - | + | - | + | - | + |
| 16 | 21 | 25 | + | +^a^ | + | + | + | + | - | + | - | + |
| 17 | 32 | 22 | + | + | + | - | + | + | + | + | - | + |
| 18 | 41 | 9 | + | + | + | + | + | + | - | - | - | + |
| 19 | 21 | 30 | + | + | + | + | + | + | + | - | - | + |
| 20 | 22 | 20 | + | NI | + | - | - | + | - | - | - | + |
| 21 | 38 | 60 | + | +^a^ | + | - | + | + | - | - | - | + |
| 22 | 22 | 30 | + | +^a^ | + | + | - | + | - | + | - | + |
| 23 | 29 | 19 | + | -^b^ | + | - | - | + | - | + | - | + |
| 24 | 29 | 22 | + | + | + | - | + | - | - | + | - | + |
| 25 | 18 | 85 | + | + | + | - | - | + | - | - | - | + |
| 26 | 18 | 38 | + | + | + | + | - | + | - | - | - | + |
| 27 | 24 | 14 | + | -^a^ | + | + | + | + | - | - | - | + |
| 28 | 22 | 35 | + | + | + | + | + | + | + | + | - | + |
| 29 | 38 | 60 | + | +^a^ | + | + | + | + | + | + | - | + |
| 30 | 31 | 40 | + | -^b^ | + | - | + | + | - | - | + | + |
| 31 | 43 | 32 | + | -^b^ | + | - | - | + | - | - | - | + |
| 32 | 26 | 18 | + | + | + | + | - | + | + | + | - | + |
| 33 | 36 | 36 | + | -^b^ | + | - | + | + | - | + | - | + |
| 34 | 25 | 35 | + | + | + | + | + | + | - | + | - | + |
| 35 | 34 | 110 | NI | + | + | + | + | + | + | + | + | + |
| 36 | 33 | 70 | NI | + | + | + | - | + | - | - | - | + |
| 37 | 19 | 54 | NI | +^a^ | + | - | + | + | - | - | - | + |

Case ID: 1-7: prepuberatal cases, 8-37: postpubertal cases, GCNIS: Germ cell neoplasm in situ, ^a^ PLAP immunohistochemistry was used, ^b^ Seminiferous tubules showed only Sertoli cells, ^c^ Testicular parenchyma was not represented in sample
